# A Comparative Analysis Between Paper-Based and Online Surveys on Parental Attitudes Towards Childhood Vaccinations

**DOI:** 10.3390/children12091161

**Published:** 2025-08-31

**Authors:** Furkan Ates, Ahmad Reza Rezaei, Julia Witkiewicz, Marta Dyszkiewicz, Dawid Lewandowski, Artur Sulik, Kacper Toczyłowski

**Affiliations:** Department of Pediatric Infectious Diseases, Medical University of Bialystok, Waszyngtona 17, 15-274 Bialystok, Poland

**Keywords:** vaccine hesitancy, health belief model, digital misinformation, cognitive bias, survey methodology, parental attitudes, childhood vaccination

## Abstract

**Highlights:**

**What are the main findings?**

- Survey method significantly influences childhood vaccination attitudes, even after adjusting for demographic and behavioral confounders.
- Online surveys show significantly lower vaccination support and higher endorsement of vaccine-skeptical views compared to paper surveys.

**What is the implication of the main findings?**

- There is a need for more tailored public health communication strategies to address online misinformation and biases.

**Abstract:**

Background/Objectives: Survey administration mode may significantly influence responses on polarizing health topics, yet this methodological factor remains understudied in vaccine hesitancy research. Understanding how data collection methods affect parental attitudes toward childhood vaccination is crucial for accurate public health surveillance and intervention design. Methods: This comparative cross-sectional study examined parental attitudes toward childhood vaccination using both paper-based (*n* = 487) and online (*n* = 386) survey administration among 873 parents. This study employed multivariate logistic regression analysis to assess differences between survey modes while controlling for demographic variables. Results: Key outcomes included general vaccination support, belief in vaccine–autism links, preference for natural immunity, and communication comfort with healthcare providers. Substantial differences emerged between survey modes. Online respondents showed significantly lower vaccination support (61.92% vs. 88.48%, *p* < 0.001), higher belief in the vaccine–autism link (37.31% vs. 16.77%, *p* < 0.001), and greater endorsement of natural immunity over vaccination (38.08% vs. 12.50%, *p* < 0.001). After adjusting for demographics, online respondents had 5 times lower odds of supporting childhood vaccination (OR = 0.20, 95% CI: 0.13–0.30) and nearly 5 times higher odds of preferring natural immunity (OR = 4.67, 95% CI: 3.19–6.95). Online respondents were also less likely to feel comfortable discussing vaccines with healthcare providers (51.0% vs. 72.7%, *p* < 0.001). Conclusions: Survey administration mode substantially influences measured parental vaccine attitudes, with online platforms capturing more vaccine-skeptical responses. These findings have critical implications for public health research methodology and suggest that mixed-mode survey designs or statistical adjustments may be necessary to obtain representative population estimates of vaccine hesitancy.

## 1. Introduction

Vaccination remains one of the most impactful public health interventions, markedly reducing childhood morbidity and mortality [1,2]. Yet the success of immunization programs increasingly hinges on parental trust and the cognitive processes that underlie vaccination decisions. Growing evidence shows that hesitancy is seldom a simple knowledge deficit; rather, it emerges from a dynamic mix of perceived susceptibility and severity, expected benefits, perceived risks, institutional trust, social norms and—critically—exposure to misinformation [3,4,5].

Large cross-national reviews and conceptual papers further demonstrate that vaccine hesitancy is a heterogeneous, context-specific phenomenon influenced by cultural, political, and informational determinants [6,7,8,9]. Understanding these determinants has become even more pressing after the COVID-19 pandemic, when parental attitudes toward routine vaccines shifted in multiple settings [10,11].

The Health Belief Model (HBM) and cognitive bias theory offer a useful lens for examining these shifts. According to the HBM, parents are more likely to vaccinate their children if they believe the disease is serious, think their child is at risk, see clear benefits to vaccination, and face few obstacles to getting the vaccine [12]. Cognitive psychology shows that people use mental shortcuts, tend to believe information that supports what they already think, and are influenced by emotions—especially when reading vaccine information online, where algorithms often show them more of what they already agree with [13,14].

While the content of online information has been widely studied, an understudied methodological factor is survey administration mode. Whether data are collected online or via paper forms may influence who responds and what they report, especially on polarizing topics [15,16]. Recent methodological work suggests that web-based surveys can oversample parents with stronger or more skeptical views, whereas paper surveys administered in healthcare settings tend to capture more conventional attitudes [17].

Building on this literature, the present study examines whether survey mode systematically affects reported parental attitudes toward childhood vaccination in Poland. We compare online and paper-based responses while controlling for key sociodemographic variables, hypothesizing that online respondents will show higher levels of vaccine hesitancy driven by cognitive, informational, and contextual factors linked to digital information consumption.

The findings are interpreted through the HBM and cognitive bias frameworks and discussed in terms of their implications for health education, survey methodology, and communication strategies designed to counter vaccine hesitancy in the digital age.

## 2. Materials and Methods

### 2.1. Study Design and Population

This cross-sectional study is a secondary analysis of data collected between March 2020 and June 2022 in the Podlaskie voivodeship of Poland.

The original questionnaire assessed guardians’ attitudes toward vaccination, including perceptions of safety, knowledge of methods to enhance immunity, views on vaccination effectiveness as a preventive measure, willingness to use non-mandatory vaccines, opinions on access to combined vaccines, awareness of side effects, and comfort in discussing vaccination with physicians. Most items were multiple-choice. Two questions employed five-point Likert scales: Question 10 (‘How much of a health threat do you consider the following infectious diseases to be?’; 1 = ‘not dangerous’ to 5 = ‘very dangerous’) and Question 11 (‘How safe do you think the following vaccines are?’; 1 = ‘not safe’ to 5 = ‘very safe’). In Question 8 (‘Where do you get your knowledge about vaccinations?’), responses were categorized as reliable or non-reliable sources. Reliable sources included healthcare professionals (e.g., doctors, pharmacists, nurses) and recognized health authorities (e.g., WHO, CDC), given their reliance on scientific evidence, regulatory oversight, and professional expertise. All other sources were classified as non-reliable. Collected demographic variables included gender, age, education, locality type, and number of children. The complete questionnaire is provided in the Supplementary Materials (Text file S1).

For this analysis, we selected 855 responses: 476 from paper-based surveys distributed to parents of hospitalized children at the Medical University of Bialystok and 379 from online respondents within the same geographic region.

Paper-based surveys were distributed to parents and legal guardians in the Department of Pediatric Infectious Diseases and our outpatient clinics. Those questionnaires were provided by trained hospital staff, who briefly explained the purpose of the study and assured participants of voluntary and anonymous participation. No personal identifiers were collected, and completed surveys were returned into sealed boxes to ensure confidentiality. Participants could ask staff members for clarification about the study before completing the survey, but responses were filled in independently without supervision. Each parent/guardian was invited to participate only once, regardless of repeat hospitalization or number of hospitalized children. If another legal guardian (e.g., grandparent) accompanied the child, they were eligible provided they were the primary caregiver making vaccination decisions. In theory, both parents/guardians of the same child could participate independently, and their responses were treated as separate since no identifying information was collected to link them.

The online survey was distributed in several Facebook groups dedicated to pediatric health and childhood vaccinations, where parents commonly discussed vaccination-related topics. These groups maintained a neutral stance toward vaccination (neither explicitly pro- nor anti-vaccine). Duplicate submissions were minimized by restricting responses to one per device and by screening for similar timestamps with identical demographic patterns.

### 2.2. Ethical Considerations

Ethical approval was granted by the Bioethics Committee of the Medical University in Bialystok (APK.002.30.2020). Participation was anonymous and voluntary. Informed consent was obtained from all participants.

### 2.3. Statistical Analysis

To guarantee the survey’s quality, we conducted two minor pilot phases, each with a sample size of 20. The first draft was sent to hospital personnel, students, and parents of hospitalized children during these phases. Based on the feedback, the questionnaire’s final version was created. Two independent individuals used Microsoft Excel to enter and compare the paper-based surveys in order to reduce mistakes. The principal investigator reviewed vague responses to determine the correct answer. Surveys with extensive missing or unreadable data, as well as those completed by respondents reporting no children, were excluded, while those with only a few missing responses were retained and accounted for during analysis. Before proceeding, each questionnaire was checked to confirm that missing data did not exceed 5%. Overall, 6.7% of paper questionnaires were excluded.

Descriptive statistics were computed for all variables. Group differences between paper and online respondents were assessed using Pearson’s chi-squared test, Fisher’s exact test, or proportion tests as appropriate. Logistic regression models were constructed to estimate the effect of survey mode on vaccine attitudes, controlling for confounders (gender, education, locality, information source reliability, and communication ease with doctors). Odds ratios (ORs) and 95% confidence intervals (CIs) were reported. All analyses were conducted in R using packages including sjPlot, gtsummary, and dplyr.

## 3. Results

### 3.1. Participant Demographics

Among the 855 respondents, 476 completed the paper survey and 379 the online version. A significantly higher proportion of online respondents were female (92.4%) compared to the paper group (84.0%). While age distribution was similar across groups, educational attainment and residence locality varied: online respondents more often had university degrees and lived in urban areas, while paper respondents were more often from rural or small-town settings. A summary of demographic differences is presented in Table 1.

### 3.2. General Attitudes Toward Vaccination

Online participants expressed markedly more hesitant attitudes across multiple indicators. Support for childhood vaccinations was reported by 89.0% of paper survey respondents but only 61.5% of online respondents. Belief in the greater danger of non-vaccination versus vaccination was similarly higher in the paper group (82.1% vs. 58.6%). Online respondents were more likely to endorse natural immunity as superior to vaccination (38.8% vs. 12.4%) and to believe in the debunked link between vaccines and autism (37.5% vs. 17.0%). These results are summarized in Table 2.

### 3.3. Information Sources and Communication Comfort

Only 21.0% of online respondents reported relying exclusively on credible medical sources, compared to 30.0% of paper respondents. Moreover, 72.5% of paper respondents indicated ease discussing vaccinations with doctors, significantly higher than the 51.2% observed in the online group. These findings are also included in Table 2.

### 3.4. Multivariate Logistic Regression Findings

Controlling for gender, education, locality, source reliability, and comfort in doctor communication, logistic regression revealed survey mode as a strong predictor of vaccine attitudes. Online respondents had significantly lower odds of supporting childhood vaccination (OR = 0.19, 95% CI: 0.12–0.28), lower belief in the risks of non-vaccination (OR = 0.35), and higher likelihood of endorsing natural immunity (OR = 4.90) and the vaccine–autism myth (OR = 2.74). These adjusted effects are presented in Table 3. These results confirm that survey format independently influences reported vaccine attitudes.

## 4. Discussion

Our results indicate that survey mode significantly influences parental vaccine attitudes. Respondents who completed the online version of the questionnaire were more likely to express vaccine hesitancy, endorse the debunked vaccine–autism link, and value natural immunity over immunization. This suggests that online environments not only reflect but may also reinforce vaccine-skeptical beliefs—likely through mechanisms such as algorithmic echo chambers and increased exposure to misinformation [13,18]. The findings of this study reveal substantial differences in vaccine attitudes based on survey mode, with online respondents displaying greater vaccine hesitancy than those completing paper-based questionnaires. This supports the hypothesis that online survey platforms may amplify certain cognitive and informational biases due to their inherent media environment [19].

From a psychological perspective, the elevated skepticism among online respondents aligns with the Health Belief Model [12] and theories of information processing. Online environments are fertile ground for echo chambers and misinformation, which reinforce existing beliefs and may increase perceived barriers to vaccination. Exposure to emotionally charged or sensationalist content—common in algorithmically curated social media—can intensify fear, mistrust, or overestimation of rare adverse events, which in turn shape vaccine decision-making [5,14,20].

People who answered the survey online were more likely to state that they trust “natural immunity” and believe the false idea that vaccines cause autism. This may be because, online, people often rely on mental shortcuts—like remembering dramatic stories about vaccine risks (availability heuristic) or searching for information that supports what they already believe (confirmation bias) [8,21,22]. These habits can lead people to misinterpret vaccine information. Online groups and social media often encourage these beliefs and lower people’s trust in vaccines, especially when group members share the same doubts or fears [23,24].

Differences in how comfortable parents feel communicating with healthcare providers may reflect varying levels of trust and engagement with the medical system. Parents who respond to paper surveys in clinical settings likely have more frequent provider contact, gaining better access to evidence-based information and supportive counseling [25,26]. This suggests that while standard outreach may be enough in healthcare environments, reaching parents who get information primarily online demands more proactive and tailored approaches [27,28].

Our findings also underscore a key methodological point: mode of survey administration can bias public health research outcomes. Online data collection may overrepresent digitally engaged or vaccine-skeptical subgroups, potentially skewing estimates of vaccine attitudes. To address this, researchers should consider mixed-mode survey designs or apply statistical adjustments to mitigate mode-related bias [15,29]. Finally, these results suggest opportunities for targeted interventions. Digital health education efforts should prioritize media literacy, engage trusted influencers, and correct misinformation with psychologically resonant narratives [30,31]. Strategies tailored for offline communities may focus more on reinforcing existing trust relationships and simplifying access to vaccine information. Future research could examine how digital information exposure interacts with cognitive traits (e.g., need for cognition, openness to experience) and assess the long-term impact of digital misinformation on vaccination behavior. Longitudinal designs could further explore whether shifts in public trust and media consumption alter vaccine confidence over time.

This aligns with recent findings from Lithuania and the Netherlands, where web-based surveys identified stronger hesitancy and shifting vaccine beliefs post-pandemic [32,33]. Similar patterns were noted in studies from the UAE, where psychological and informational determinants of vaccine decisions were found to be shaped heavily by digital trust and parental anxiety [3,34].

The results also support the HBM, particularly the role of perceived susceptibility and perceived barriers in shaping vaccine-related behaviors [12]. Trust in health authorities and credible media outlets plays a mediating role, as shown in comparative research across multiple countries [19]. The divergence in attitudes between online and paper respondents in our study may be partially explained by differing exposure to online content and divergent media consumption habits—factors that have become increasingly relevant during and after the COVID-19 pandemic [35,36].

However, our study also has several limitations that must be acknowledged, which could affect the generalizability and interpretation of the results.

The use of self-reported data, which introduces response bias, is one of the main limitations. It is possible for participants to give replies that are socially acceptable or that they do not remember their own choices and views. This restriction is frequently mentioned in survey-based research, as demonstrated by a prior Italian study from 2020 that addressed related issues in their investigation of occupational physicians’ opinions toward the TBE vaccine [37].

It is also not possible to completely rule out a considerable selection bias in the paper-based surveys. Similar to the online surveys, actively participating in person in our department may also reflect an intense or passionate attitude toward vaccines.

Additionally, the sample showed demographic imbalances, particularly in age and sex distribution, which may limit representativeness. For example, mothers make up the majority of the sample, with more than 90% of the responders being female. Although demographic factors were controlled for in the analysis, unmeasured confounding cannot be excluded.

Another limitation of this study is that response rates and refusal data were not recorded, which prevents us from quantifying potential non-response bias. This should be considered when interpreting the representativeness of the findings.

Furthermore, the reproducibility of this study in other settings may be influenced by cultural norms, trust in healthcare systems, and country-specific vaccination policies, which could affect how parents respond to the same instrument. Although the questionnaire itself can be replicated, caution should be exercised when interpreting cross-country comparisons.

Finally, although the observed correlations point to a relationship between vaccine safety assessment and knowledge gaps as well as overall attitudes about vaccinations, it is impossible to definitively determine the cause because of the possibility of unidentified causes. Similar findings were reached by prior research [38].

This research adds to growing evidence suggesting that survey methodology must account for digital bias, especially in studies of vaccine behavior. Mixed-mode or hybrid surveys could offer more accurate representations of public attitudes. Moreover, communication-based interventions have been effective in adjusting perceptions and hesitancy when healthcare professionals are trained in motivational strategies and empathy [39,40]. Furthermore, a systems-level approach to analyzing vaccine data, as suggested in vaccinomics and big data literature, may help monitor real-time changes in hesitancy patterns [41,42].

## 5. Conclusions

This study highlights that the method of survey administration—online vs. paper—can significantly influence reported parental attitudes toward childhood vaccination. Online respondents showed greater vaccine hesitancy, more belief in misinformation, and less trust in healthcare communication. These differences persisted even when controlling for key demographic and behavioral variables, underscoring the independent effect of survey mode. The findings support the need for psychological awareness in survey design and public health outreach, especially in digital contexts prone to cognitive biases and echo chambers. Future health promotion strategies must account for the influence of information environments on vaccine attitudes and should tailor communication accordingly to reduce vaccine hesitancy.

## Figures and Tables

**Table 1:** Demographic profile of respondents by survey type.

Characteristic	N	Survey Type		*p*
		Online, *n* = 379 ^a^	Paper, *n* = 476 ^a^	
Gender:	849			<0.001 ^b^
female		350 (92.4)	395 (84.0)	
male		29 (7.65)	75 (15.96)	
Age:	846			0.332 ^c^
up to 29 years		61 (16.14)	82 (17.6)	
30–39 years		232 (61.2)	279 (59.7)	
40–49 years		73 (19.3)	98 (21.0)	
50–59 years		11 (2.9)	7 (1.5)	
60 years or above		2 (0.5)	1 (0.2)	
Education:	848			<0.001 ^c^
high		314 (82.9)	328 (69.9)	<0.001 ^d^
vocational		4 (1.0)	38 (8.1)	<0.001 ^d^
primary and secondary		61 (16.1)	103 (22.0)	0.034 ^d^
Locality:	842			<0.001 ^b^
big city (over 300,000 inhabitants)		134 (35.4)	111 (24.0)	<0.001 ^d^
city (up to 300,000 inhabitants)		146 (38.5)	141 (30.5)	0.007 ^d^
town (up to 30,000 inhabitants)		49 (12.9)	111 (24.0)	<0.001 ^d^
village		50 (13.2)	100 (21.6)	0.004 ^d^
Number of children	842			0.725 ^b^
one or two		304 (81.1)	383 (82.0)	
three or above		71 (18.9)	84 (18.0)	

^a^ *n* (%); ^b^ Pearson’s chi-squared test; ^c^ Fisher’s exact test; ^d^ proportion test.

**Table 2:** Analysis of the general attitudes towards vaccination by survey type.

Characteristic	N	Survey Type		*p* ^b^
		Online, *n* = 379 ^a^	Paper, *n* = 476 ^a^	
General attitude toward vaccination				
Support for childhood vaccinations	854	233 (61.5)	423 (89.0)	<0.001
Belief that lack of childhood vaccination poses greater risk	853	222 (58.6)	389 (82.1)	<0.001
Support for natural immunity over childhood vaccinations	848	147 (38.8)	58 (12.4)	<0.001
Favoring free highly combined vaccines (e.g., 6-in-1)	854	171 (45.1)	292 (61.5)	<0.001
Belief in vaccine–autism link	851	142 (37.5)	80 (17.0)	<0.001
Factors influencing public health perceptions and behaviors				
Using only reliable vaccine information sources	855	79 (20.8%)	143 (30.0)	0.002
Having no issues discussing vaccinations with the doctor	848	194 (51.2)	340 (72.5)	<0.001

^a^ *n* (%); ^b^ proportion test.

**Table 3:** The adjusted exposure effects of the fitted multivariate logistic regression.

Outcome Variable	Adjusted Exposure Effect (Online Survey with Reference to Paper One)		
	OR	CI 95%	*p*
Occurrence of supporting childhood vaccination	0.19	0.12–0.28	<0.001
Occurrence of belief that lack of childhood vaccination poses greater risk	0.35	0.24–0.49	<0.001
Occurrence of supporting natural immunity over childhood vaccinations	4.90	3.32–7.32	<0.001
Occurrence of favoring free highly combined vaccines (e.g., 6-in-1)	0.53	0.39–0.72	<0.001
Occurrence of believing in vaccine–autism link	2.74	1.93–3.92	<0.001

## Data Availability

The raw data supporting the conclusions of this article will be made available by the corresponding authors upon reasonable request.

## Supplementary Materials

The following supporting information can be downloaded at: https://www.mdpi.com/article/10.3390/children12091161/s1, Text file S1: The complete questionnaire used in this study, translated to English.

## Abbreviations

The following abbreviations are used in this manuscript:

HBM	Health Belief Model
OR	Odds ratio
CI	Confidence interval
